# Intra-abdominal hypertension and reverse Trendelenburg position increase frontal QRS-T angle in laparoscopic cholecystectomy: An observational study

**DOI:** 10.1097/MD.0000000000041934

**Published:** 2025-03-14

**Authors:** Ali Genç, Uğur Özsoy, Ahmet Tuğrul Şahin, Mehtap Gürler Balta, Vildan Kölükçü, Gülşen Genç Tapar, Tuğba Karaman, Serkan Karaman

**Affiliations:** a Anesthesiology and Reanimation Department, Tokat Gaziosmanpasa University Hospital, Tokat, Turkey; b General Surgery Department, Tokat Gaziosmanpasa University Hospital, Tokat, Turkey; c Cardiology Department, Tokat Gaziosmanpasa University Hospital, Tokat, Turkey.

**Keywords:** cardiac arrhythmias, electrocardiography, general anesthesia, pneumoperitoneum, vectorcardiography

## Abstract

Increased intra-abdominal pressure during laparoscopic surgery, anesthesia, patient position, and neuroendocrine response may increase the risk of arrhythmia. This study aimed to investigate the perioperative changes in the frontal QRS-T angle in patients undergoing laparoscopic cholecystectomy under general anesthesia. Therefore, electrophysiological parameters at different stages of laparoscopic cholecystectomy were studied using the frontal QRS-T angle and the risk of arrhythmia susceptibility was investigated. This prospective observational study included 48 patients aged 23 to 65 years with an American Society of Anesthesiologists score of 1 to 3 who underwent laparoscopic cholecystectomy in the operating room of Gaziosmanpaşa University Research and Application Hospital. Electrocardiographic recordings were obtained immediately before surgery, immediately before and after intra-abdominal carbon dioxide insufflation, 2 minutes after reverse Trendelenburg, immediately after extubation, and 2 hours postoperatively, and the frontal plane QRS-T angle, QT and QTc interval were studied. Rhythm disturbances, bleeding and complications were recorded. The frontal QRS-T angle, QT and QTc interval were significantly increased with intra-abdominal hypertension (IAH) compared to baseline (*P* < .001, *P* < .001, *P* < .001, respectively). Similarly, frontal QRS-T angle, QT, and QTc interval increased significantly with reverse Trendelenburg position compared to baseline (*P* < .001, *P* < .001, *P* < .001, respectively). The frontal QRS-T angle, which increased with IAH and the reverse Trendelenburg position, significantly decreased immediately after the patient woke up (*P* < .001). Heart rate and mean arterial pressure increased significantly with IAH compared to those just before carbon dioxide insufflation (*P* = .03, *P* < .001, respectively). The present study found that IAH induction and reverse Trendelenburg positioning increased the frontal QRS-T angle, QT, and QTc interval in patients undergoing laparoscopic cholecystectomy. These prolonged values may cause serious arrhythmias, particularly in patients with cardiac disease. Therefore, it is very important for anesthetists to be aware of electrocardiographic changes such as arrhythmias in patients undergoing laparoscopic cholecystectomy.

## 1. Introduction

Cholecystectomy is a surgical procedure performed under various conditions such as obstruction of the biliary tract by stones, porcelain gallbladder or acalculous cholecystitis. Although laparoscopic surgery can be performed open or laparoscopically, it has been preferred in recent years due to its advantages.^[1]^ Laparoscopic surgery is performed by placing a needle or trocar intraabdominally and reaching a certain intra-abdominal pressure (IAP) in the abdominal cavity by carbon dioxide insufflation. Physiologically, IAP ranges from 0 to 7 mm Hg and a pressure increase >12 mm Hg is defined as intra-abdominal hypertension (IAH). Carbon dioxide (CO2) is an ideal gas because it has low flammability and high blood solubility, which reduces the risk of gas embolism.

Laparoscopic cholecystectomy has been shown to have advantages over open surgery, including less postoperative pain, early onset of bowel movements, shorter hospital stay, earlier return to daily activities and better aesthetic results.^[1]^ However, an increased IAP may adversely affect the respiratory, circulatory, neuroendocrine and central nervous systems. An increased IAP may compress the vena cava and abdominal aorta and cause impaired perfusion of other organs, especially the kidneys and spleen.^[2]^ In addition, this pathologic process decreases cardiac preload, stroke volume, and cardiac output and increases central venous pressure, pulmonary capillary wedge pressure, pulmonary artery pressure and left ventricular afterload.^[3]^ IAH may also impair cardiac perfusion by affecting pressure in the coronary arteries.^[2]^ It should be taken into consideration that increased IAP has also been defined as an independent risk factor for mortality in critically ill patients.^[3,4]^

The QT interval, which expresses the time required for ventricular depolarization and repolarization, is the interval from the beginning of the QRS complex to the end of the T wave. Because the QT interval is affected by heart rate, QT corrected for heart rate is called QTc. Perioperative prolongation of QT and QTc intervals may result in serious complications including severe arrhythmias, ventricular tachycardia, ventricular fibrillation, and cardiac arrest.^[5]^ Variable QT intervals have been associated with heterogeneous repolarization and ventricular arrhythmias.^[6]^

The frontal QRS-T angle is expressed as the absolute difference between the QRS and T wave axes. The frontal QRS-T angle, a parameter that can be easily calculated from a 12-lead surface electrocardiography (ECG), is considered to be a marker of ventricular repolarization heterogeneity.^[7,8]^ The frontal QRS-T angle provides important information about the clinical conditions of diseases such as heart failure, obesity, and acute coronary syndrome.^[8,9]^ Increased ventricular repolarisation heterogeneity is associated with an increased risk of arrhythmogenesis. Studies have shown that an increased frontal QRS-T angle increases the risk of cardiovascular and arrhythmic events and is associated with increased mortality risk.^[7]^ Previous research has suggested that the frontal QRS-T angle provides a more robust and reproducible measure of ventricular repolarization than the QT/QTc interval, as it is less influenced by external factors.^[10]^ A wider frontal ORS-T angle is considered a strong and independent risk factor for cardiac morbidity and mortality, surpassing other traditional cardiovascular risk factors and electrocardiographic markers, such as QT interval length.^[10,11]^ In addition, some values obtained from ECG have been reported to be associated with the severity of ischemic stroke, the presence of myocardial fibrosis, and the circadian course of hypertension.^[12–14]^

Increased IAP, anesthetic drugs, positions given to the patient and neuroendocrine response in laparoscopic surgeries may increase the risk of arrhythmia. There are many studies on the effects of laparoscopic surgeries that increase IAP, anesthetic drugs and anesthesia methods on QT/QTc duration, but there are a limited number of studies in the literature on the effects of another repolarization parameter, the frontal QRS-T angle. The present study aimed to investigate peroperative frontal QRS-T angle changes in patients undergoing laparoscopic cholecystectomy under general anesthesia. Therefore, the risk of arrhythmia susceptibility was investigated at different stages of laparoscopic cholecystectomy using the QRS-T angle in the frontal plane.

## 2. Materials and methods

This study was conducted in patients undergoing laparoscopic cholecystectomy under general anesthesia in the operating room of the Tokat Gaziosmanpaşa University Health Research and Application Center after approval of the Tokat Gaziosmanpaşa University Clinical Research Ethics Committee (24-KAEK-215) and ClinicalTrials.gov (NCT06651450). The patients were informed about the study before surgery and written informed consent was obtained from all patients. Between August 2024 and January 2025, a total of 48 patients aged 23 to 65 years with an American Society of Anesthesiologists (ASA) score of 1 to 3 were included in the study. Patients with severe cardiovascular diseases (coronary artery disease, atrial fibrillation, atrial flutter, heart failure, pacemaker, and bundle branch block), poor aerobic exercise capacity (lower than 4 metabolic equivalents), those taking drugs that may prolong electrocardiographic intervals such as QT and QTc, respiratory diseases, metabolic diseases, vascular diseases, renal failure, electrolyte disturbances, or severe psychiatric disorders were excluded from the study. Demographic data (age, sex, ASA score, body mass index, and complications) were recorded.

Patients were taken to the operating room and routine ECG, noninvasive blood pressure, pulse, and oxygen saturation monitoring (CARESCAPE™ B650, GE Healthcare, USA) was performed. The patient’s basal fluid requirement, calculated for the period after discontinuing oral fluid intake, was met preoperatively with an intravenous balanced electrolyte solution. Before induction of anesthesia, a mask providing 100% oxygen was placed on the patient’s face without air leakage and the patient was preoxygenated by breathing at a normal tidal volume for 3 minutes. Anesthesia was induced with 2 mg/kg propofol intravenously (iv), fentanyl 1 µg/kg iv, rocuronium 0.6 mg/kg iv. Patients were ventilated with a mask for at least 2 minutes after administration of a neuromuscular blocker, and intubated and anesthesia was maintained with inhaled sevoflurane at 1 minimum alveolar concentration. Hemodynamic values were maintained within ±20% of the baseline values. End-tidal carbon dioxide values were maintained at 35 ± 3 throughout the operation. When the operation was completed, sevoflurane was stopped and neuromuscular blockade was reversed using 2 mg/kg sugammadex iv. In all patients, 12-channel ECG (Cardiofax M ECG-3350, Nihon Kohden, Tokyo, Japan) recordings at 25 mm/s and 10 mm/mV were obtained immediately before induction of anesthesia (T1), immediately before (T2), and after (T3) intra-abdominal CO2 insufflation, 2 minutes after reverse Trendelenburg (T4) and immediately after extubation (T5), and 2 hours after surgery (T6). The primary outcome was frontal QRS-T angle, while the secondary outcomes were QT and QTc interval, hemodynamic change, complication, and rhythm disturbance.

**Figure 1.**
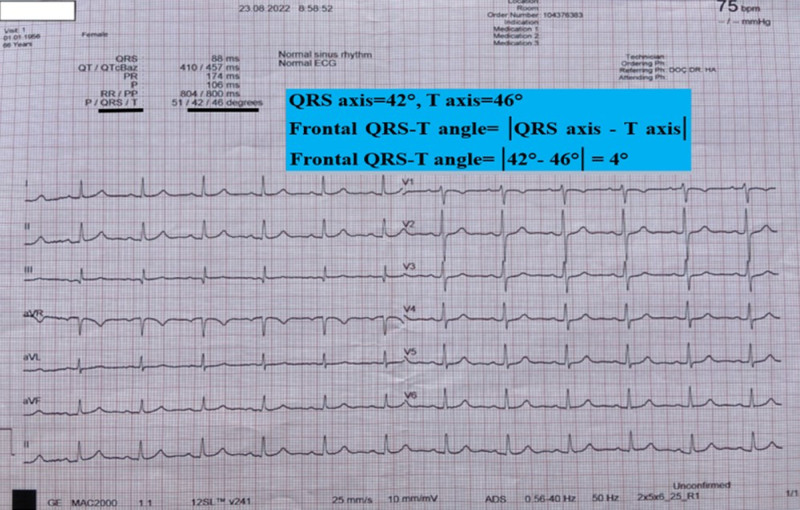
Frontal QRS-T calculation with QRS axis and T axis obtained from automatic report section of 12-lead ECG device. 12-channel ECG recording at 25 mm/s and 10 mm/mV, | | = absolute value.

The frontal QRS-T angle is expressed as the absolute difference between the QRS and T wave axes (Fig. 1). The QT interval is the time interval from the beginning of the QRS complex to the end of the T wave. Since the QT interval is affected by the heart rate, it is called QT corrected for the heart rate, QTc interval. QT and QTc intervals are given directly on the 12-lead surface ECG (Fig. 1).

### 2.1. Statistical analysis

Statistical conformity of the data to a normal distribution was evaluated using one-sample Kolmogorov–Smirnov test. Qualitative data were expressed as numbers and percentages, normally distributed quantitative data as mean and standard deviation, and non-normally distributed quantitative data as median (minimum–maximum) values. Repeated measures data were evaluated with repeated measures ANOVA test when normally distributed and Friedman two-way ANOVA test when not normally distributed. post hoc Bonferroni corrections were applied for pairwise comparisons. The Statistical Package for Social Sciences (Version 20.0, SPSS Inc., Chicago, IL) was used to evaluate all data. Statistical significance was set at *P* < .05.

When the sample size was calculated and; the effect size obtained from a previous study^[2]^ was accepted as 0.4, the type 1 error value was 0.05, and the power was 0.80, it was determined that the sample size for the study was sufficient with 44 patients. Considering the losses that may occur during follow-up, the study was planned to be conducted with 48 patients with an increase of 10%.

## 3. Results

**Figure 2.**
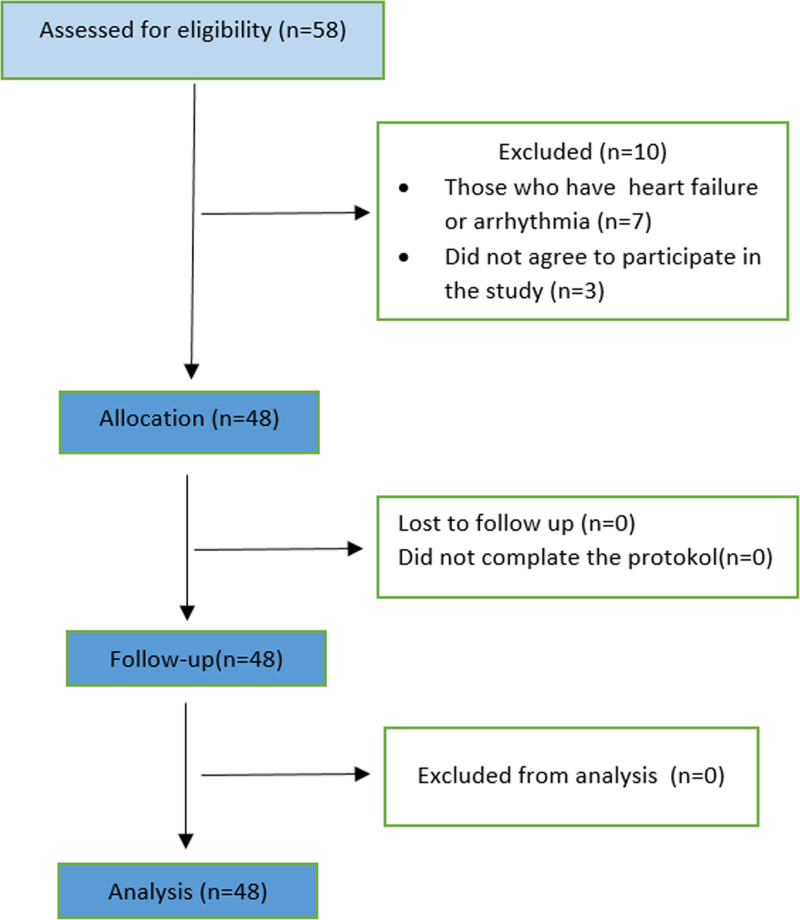
Flow diagram of the study.

Fifty-eight patients were included in this study. Seven patients were ineligible, and 3 patients did not want to participate in the study. A total of 48 patients were included in the study, and no patients were excluded. A flow chart of the study is shown in Figure 2.

Demographic data of the patients are presented in Table 1. Of the 48 patients aged between 23 and 65 years, 28 were female and 20 were male. The body mass index of the patients ranged from 18.26 to 36.39 with a median value of 28.42. Of the 48 patients, 12 had ASA1, 30 had ASA2 and 6 had ASA3. Of all patients, 16 had hypertension, 10 had diabetes mellitus, 2 had chronic obstructive pulmonary disease, and 20 had no comorbidities.

**Table 1:** Demographic data.

Age (years): median (min–max)	51 (23–65)
Gender (male/female): (n)	20/28
BMI (kg/m^2^): median (min–max)	28.42 (18.26–36.39)
ASA score: (I/II/III) No comorbid diseases: (n) Hypertension: (n) Diabetes: (n) Chronic lung disease: (n)	12/30/6 20 16 10 2

ASA = American Society of Anesthesiologists, BMI = body mass index, max = maximum, min = minimum, n = number.

**Figure 3.**
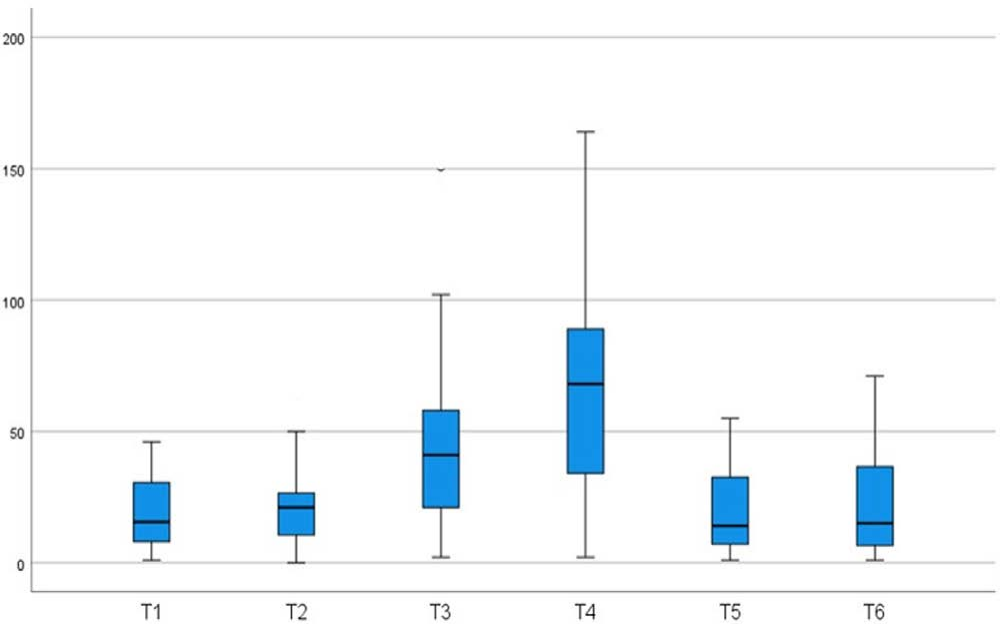
Changes on frontal QRS-T angle at different stages of surgery. T1 = immediately before induction of anesthesia, T2 = immediately before CO2 insufflation, T3 = immediately after CO2 insufflation, T4 = 2 min after reverse Trendelenburg position, T5 = immediately after patient extubation, T6 = 2 h after patient extubation.

There was a statistically significant difference in the frontal QRS-T angle at the different stages of surgery (Table 2). The frontal QRS-T angle increased significantly with IAH compared to the baseline (T1) value (*P* < .001). Similarly, the frontal QRS-T angle increased significantly more in the reverse Trendelenburg position compared to the baseline value (*P* < .001). There was a statistically significant increase in the frontal QRS-T angle from T2 to T3 and a statistically significant decrease from T4 to T5 (*P* < .001, *P* < .001, respectively). The frontal QRS-T angle returned to baseline after extubation (T5). There were no statistically significant differences in the frontal QRS-T angle from T1 to T2, T3 to T4 and T5 to T6. Changes in the frontal QRS-T angle at different stages of the surgery are shown in Figure 3.

**Table 2:** Values of monitored parameters at certain stages of surgery.

	T1	T2	T3	T4	T5	T6	*P*
Heart rate (beats/min) Mean ± SD	72.90 ± 8.36	76.21 ± 11.30	83.50 ± 12.78	80.73 ± 16.47	77.98 ± 13.88	74.37 ± 11.36	<.001*^,^†
MAP (mm Hg) Mean ± SD	84.23 ± 5.85	77.60 ± 5.84	82.90 ± 5.27	75.94 ± 5.84	84.81 ± 4.25	83.29 ± 5.17	<.001*^,^†
fQRS-T angle Median (min–max)	15.50 (1–65)	21 (0–60)	41 (2–151)	68 (2–164)	14 (1–80)	15 (1–71)	<.001*^,b^
QT interval Median (min–max)	381 (280–452)	389 (342–604)	416 (328–610)	434 (282–638)	419 (346–576)	385 (332–448)	<.001*^,^‡
QTc interval Median (min–max)	416.5 (315–486)	423.5 (374–640)	445.5 (388–655)	456 (358–657)	445 (395–607)	415.5 (356–481)	<.001*^,^‡
PR interval Median (min–max)	150 (108–278)	149 (98–336)	155 (94–301)	158 (92–310)	156 (92–288)	154 (106–228)	.037*^,^‡

MAP = mean arterial pressure, max = maximum, min = minimum, SD = standard deviation, T1 = immediately before induction of anesthesia, T2 = immediately before CO2 insufflation, T3 = immediately after CO2 insufflation, T4 = 2 min after reverse Trendelenburg position, T5 = immediately after patient extubation, T6 = 2 h after patient extubation.

* Statistically significant.

† Repeated measures ANOVA test.

‡ Friedman two-way ANOVA test.

There was a statistically significant difference in the QT interval at different stages of surgery (Table 2). The QT interval increased significantly with IAH compared to the baseline (T1) value (*P* < .001). Similarly, the QT interval was significantly increased in the reverse Trendelenburg position compared to the baseline value (*P* < .001). A statistically significant increase in QT interval was observed in the transition from T2 to T3 (*P* = .018). There was a statistically significant decrease in the QT interval from T5 to T6 (*P* = .018). The QT interval returned to baseline 2 hours after extubation (T6). There was no statistically significant difference in the QT interval from T1 to T2, T3 to T4, and T4 to T5.

There was a statistically significant difference in the QTc interval at different stages of surgery (Table 2). The QTc interval increased significantly with IAH compared with the baseline (T1) value (*P* < .001). Similarly, the QTc interval increased significantly in the reverse Trendelenburg position compared to baseline (*P* < .001). A statistically significant increase in the QTc interval was observed during the transition from T2 to T3 (*P* = .04). There was a significant decrease in the QTc interval from T5 to T6 (*P* < .001). The QTc interval returned to baseline 2 hours after extubation (T6). There was no statistically significant difference in the QTc interval from T1 to T2, T3 to T4, and T4 to T5.

There was a statistically significant difference in the mean arterial pressure at different stages of surgery (Table 2). The mean arterial pressure decreased significantly from T1 to T2 and from T3 to T4 (*P* < .001, *P* < .001, respectively). The mean arterial pressure increased significantly from T2 to T3 and from T4 to T5 (*P* < .001, *P* < .001, respectively). There was no statistically significant difference in the mean arterial pressure from T5 to T6.

There was a statistically significant difference in heart rate at different stages of surgery (Table 2). There was a statistically significant increase in the heart rate from T2 to T3 (*P* = .03). There were no statistically significant differences in heart rate from T1 to T2, T3 to T4, T4 to T5, and T5 to T6. In addition, in this study, ventricular extrasystole was observed in 4 patients and atrial extrasystole in 2 patients after CO2 insufflation, which did not disrupt hemodynamics, did not require any intervention, and disappeared spontaneously in the intraoperative period.

## 4. Discussion

This study showed that IAH triggered by CO2 insufflation and reverse Trendelenburg position increased the frontal QRS-T angle, QT, and QTc interval in patients undergoing laparoscopic cholecystectomy, and these values decreased after extubation and 2 hours after surgery.

Although changes in cardiac function caused by IAH have been investigated in many studies, more studies investigating their effect on ECG parameters are needed.^[2]^ Elevation of the diaphragm may cause some differences in ECG variables by changing the cardiac electrical axis.^[15]^ Increased intrathoracic pressure due to diaphragm elevation triggered by IAH may cause cardiac dectrocardia and decrease in diastolic function by decreasing ventricular compliance.^[16]^ IAH may impair systolic function by decreasing the cardiac contractility.^[16,17]^ In addition, increased IAP may impair cardiac nutrition by decreasing coronary artery perfusion pressure.^[18]^ These effects of IAH may also cause changes in ECG parameters.

The frontal QRS-T angle is a powerful electrocardiographic parameter for predicting arrhythmia risk and cardiovascular mortality.^[10,11]^ Dabrowski et al showed that IAH increased spatial QRS-T angle and this increased angle decreased with Trendelenburg position in a study performed in laparoscopic gynecology operations.^[2]^ They stated that the decrease in spatial QRS-T angle in the Trendelenburg position may be related to an increase in end-diastolic volume and improvement in systolic function such as the left ventricle and cardiac output.^[2,19]^ The present study found that the frontal QRS-T angle increased in IAH. The further increase in the frontal QRS-T angle with the reverse Trendelenbug position compared to the baseline ECG may be related to a further decrease in systolic function such as end-diastolic volume, left ventricle and cardiac output. Stroke volume, which has a strong effect on atrial volume, is a sensitive predictor of ventricular repolarization disorders.^[20,21]^ Therefore, both IAH and reverse Trendelenburg positioning in laparoscopic cholecystectomy may impair ventricular repolarization, however, this needs to be investigated in more studies.

QT, which corresponds to ventricular depolarization and repolarization, and QTc interval reflecting electrical instability in the ventricles, has been shown to be associated with cardiac arrhythmias and mortality.^[5,6,22]^ Ekici et al showed that high IAP increased QT and QTc interval more than a low IAP in laparoscopic cholecystectomy operations.^[23]^ Therefore, they recommended that low IAP should be preferred in laparoscopic surgery especially in patients with cardiac disease. In another study, Egawa et al showed that IAP increased QT and QTc intervals in both young and elderly patients, but long-term IAP caused a more significant increase in the elderly.^[22]^ In the present study, it was found that IAH increased QT and QTc interval, and these values increased even more with the reverse Trendelenbug position given to the patient compared to the baseline values.

Regarding perioperative hemodynamic variables, Dabrowski et al reported in their study performed in laparoscopic gynecological surgery that the mean heart rate increased in IAH compared to the preoperative period, and the mean heart rate decreased slightly in the Trendelenburg position.^[2]^ They also showed that the mean arterial pressure decreased in the IAH compared to the preoperative period, and the mean arterial pressure increased slightly in the Trendelenburg position. Ekici et al reported an increase in mean arterial pressure and heart rate with CO2 insufflation during laparoscopic cholecystectomy performed on a neutral operating table.^[23]^ In the present study, the mean arterial pressure and heart rate decreased preoperatively before CO2 insufflation, increased slightly after CO2 insufflation, decreased in the reverse Trendelenburg position and returned to baseline levels postoperatively.

Many factors such as anesthetic agents administered in patients undergoing surgery can affect ECG variables. Intravenous anesthetics, inhaled anesthetics, and analgesics such as opioids used in anesthesia may affect ECG variables such as the frontal QRS-T angle. Sevoflurane prolongs the QTc interval while propofol shortens this interval.^[24]^ Desflurane may increase the frontal QRS-T angle depending on the flow rate.^[25]^ However, Abrich et al reported that propofol infusion does not affect the QTc interval.^[26]^ Moreover, Tascanov et al showed that propofol prolonged the frontal QRS-T angle and QT, QTc, and Tp-e intervals.^[8]^ It has been shown that opioids used in the administration of anesthesia can prolong the QTc interval depending on the dosage.^[27,28]^ It has also been reported that spinal anesthesia increases the frontal QRS-T angle and this increase is greater in obese patients.^[29]^ In addition, a significant relationship was found between diurnal blood pressure change and frontal QRS-T angle in hypertension, which is a leading cause of cardiovascular morbidity and mortality.^[14]^ The frontal QRS-T angle was found to be high in those whose blood pressure did not decrease at night or was high.^[14]^

The present study has several limitations. In this study, propofol was used for induction of anesthesia, sevofluorane for maintenance of anesthesia, and opioids for analgesia, and these may have an effect on changes in ECG variables in addition to IAH and reverse Trendelenburg position. However, to minimize this effect, ECG recordings were taken immediately before and after CO2 insufflation and 2 minutes after reverse Trendelenburg position. In addition, patients with hypertension, which may increase the frontal QRS-T angle, and patients with blood pressure that does not decrease or is high at night (reverse dipping) could not be identified in our study. Additionally, in the present study, the effects of high IAP and reverse Trendelenburg position on the frontal QRS-T angle were investigated, whereas the effects of low IAP and other positions on this angle were not evaluated. Therefore, further studies are needed to investigate the effects of low IAP and other positions on the frontal QRS-T angle.

In conclusion, the frontal QRS-T angle at can be simply calculated from the ECG and provides important information about myocardial depolarization and repolarization. In patients undergoing laparoscopic cholecystectomy, IAH and reverse Trendelenburg positioning may be associated with increased frontal QRS-T angle, QT, and QTc intervals. Because these prolonged values may cause serious arrhythmias, especially in patients with cardiac disease, close monitoring of patients undergoing laparoscopic cholecystectomy and taking necessary precautions are very important for safe and effective anesthesia.

## Author contributions

**Investigation:** Ali Genç.

**Methodology:** Ali Genç, Gülşen Genç Tapar.

**Resources:** Ali Genç, Mehtap Gürler Balta.

**Software:** Ahmet Tuğrul Şahin, Mehtap Gürler Balta, Vildan Kölükçü.

**Validation:** Ali Genç, Uğur Özsoy, Ahmet Tuğrul Şahin, Mehtap Gürler Balta, Vildan Kölükçü, Tuğba Karaman, Serkan Karaman.

**Visualization:** Ali Genç.

**Writing – original draft:** Ali Genç.

**Writing – review & editing:** Ali Genç, Uğur Özsoy, Vildan Kölükçü, Gülşen Genç Tapar, Tuğba Karaman, Serkan Karaman.
